# Low appendicular skeletal muscle index increases the risk of carotid artery plaque in postmenopausal women with and without hypertension/hyperglycemia: a retrospective study

**DOI:** 10.1186/s12877-023-04076-w

**Published:** 2023-06-20

**Authors:** Yayun Lu, Jianguang Tian, Liangyu Wu, Qing Xia, Qinzhong Zhu

**Affiliations:** 1 Health Examination Center, Huadong Sanatorium, No.67 Jinyuan Road, Wuxi, 214065 People’s Republic of China; 2 Department of Science and Education, Huadong Sanatorium, No.67 Jinyuan Road, Wuxi, 214065 People’s Republic of China

**Keywords:** Appendicular skeletal muscle index, Carotid artery plaque, Postmenopausal women, Hyperglycemia, Hypertension

## Abstract

**Background:**

This study aimed to evaluate whether the low appendicular skeletal muscle index (ASMI) is closely associated with the risk of carotid artery plaque (CAP) in postmenopausal women with and without hypertension/hyperglycemia stratified by body mass index (BMI) categories.

**Methods:**

A total of 2048 Chinese postmenopausal women aged 40–88 years were eventually enrolled in this retrospective study. Skeletal muscle mass was estimated by using segmental multifrequency bioelectrical impedance analysis. ASMI was defined as follows: appendicular skeletal muscle mass(kg)/[height(m)]^2^. CAP was assessed by B-mode ultrasound. We explored the association between ASMI quartiles or low skeletal muscle mass and the risk of CAP by using multivariate-adjusted logistic regression models. A potential nonlinear relationship was also tested using restricted cubic spline regression.

**Results:**

CAP was observed in 289/1074 (26.9%) normal-weight and 319/974 (32.8%) overweight/obese postmenopausal women. Individuals with CAP had significantly lower ASMI values than those without (*P* < 0.001). The ASMI value also showed a linear relationship with the CAP risk in postmenopausal women stratified by BMI category (*P*_for non−linearity_ > 0.05). In comparison with the highest ASMI quartile, the lowest ASMI quartile was significantly associated with a high risk of CAP development in non-hypertensive individuals with normal weight (odds ratio [OR] = 2.43; 95% confidence interval [CI]: 1.44 ~ 4.12) or overweight/obesity (OR = 4.82, 95% CI: 2.79 ~ 8.33), hypertensive individuals with normal weight (OR = 5.90, 95% CI: 1.46 ~ 11.49) or overweight/obesity (OR = 7.63, 95% CI: 1.62 ~ 35.86), non-hyperglycemic individuals with normal weight (OR = 2.61, 95% CI: 1.54 ~ 4.43) or overweight/obesity (OR = 2.94, 95% CI: 1.84 ~ 4.70), and hyperglycemic individuals with normal weight (OR = 6.66, 95% CI: 1.08 ~ 41.10) or overweight/obesity (OR = 8.11, 95% CI: 2.69 ~ 24.49). Moreover, low skeletal muscle was independently associated with the risk of CAP in postmenopausal women, regardless of the BMI category.

**Conclusion:**

ASMI was inversely associated with the risk of CAP development in postmenopausal women, especially in patients with high blood sugar and/or hypertension, indicating that skeletal muscle mass maintenance may contribute to prevention of CAP in postmenopausal women.

**Supplementary Information:**

The online version contains supplementary material available at 10.1186/s12877-023-04076-w.

## Background

Sarcopenia is a chronic disease characterized by progressive reductions in skeletal muscle mass, muscle strength, and/or muscle function with advancing age, which contribute to muscle weakness and a decline in physical function. The prevalence of sarcopenia has been increasing, causing serious health problems in the elderly population [1, 2]. Accumulating evidence has confirmed that sarcopenia is significantly associated with metabolic abnormalities, especially in patients with type 2 diabetes mellitus [3–6].

Diabetes increases the risk of atherosclerotic cardiovascular diseases and is associated with high rates of morbidity and mortality [7]. Atherosclerosis is a chronic process involving both innate and acquired immune responses that leads to cardiovascular diseases [8, 9]. Carotid artery plaque (CAP) progression is also associated with a higher risk of developing cardiovascular events [10, 11], Previous studies have indicated that individuals with diabetes showed significantly greater carotid intima-media thickness (cIMT) than non-diabetic individuals [12]. Moreover, hypertensive individuals are at a higher risk of developing CAP due to the positive effect of hypertension in promoting vascular sclerosis. Therefore, both hyperglycemic and hypertensive populations deserve more attention in efforts to prevent CAP [13, 14].

Recent studies have illustrated that skeletal muscle mass is negatively related to subclinical atherosclerosis based on artery calcification scores in elderly patients [15, 16], while another study also showed that lower thigh muscle mass increased the risk of atherosclerosis development assessed by cIMT among older males [17, 18]. Since the prevalence of metabolic syndrome in postmenopausal women was higher than that in men, ranging from 32 to 58%, and metabolic syndrome became a combination of risk factors that increased the risk of cardiovascular diseases [19, 20]. However, studies on the association between low appendicular skeletal muscle index (ASMI) and the risk of CAP in postmenopausal women stratified by different body mass index (BMI) categories are still lacking, and this association has not been explored in hyperglycemic or hypertensive individuals either. Therefore, we evaluated the BMI-specific associations between low ASMI and the risk of CAP in individuals with and without hyperglycemia or hypertension.

## Methods

### Study population

We performed a retrospective analysis of Chinese postmenopausal women undergoing annual health examinations in Huadong Sanatorium of Wuxi city, China, from January 2021 to December 2021. All participants had experienced no spontaneous menses for more than one year. A total of 2048 participants aged 40–88 years who had undergone carotid ultrasonography and body composition analysis and had data available for all covariates were finally included in this study. We excluded participants with incomplete medical records, surgical menopause, hormone replacement therapy, malignancy, or amputated extremities. The mean age (± standard deviation) of all included participants was 57.25 ± 7.54 years. Since most postmenopausal women in our study had no history of smoking and drinking, data on cigarette use and alcohol consumption were not included in the statistical analysis. This study was performed in accordance with the principles of the Declaration of Helsinki and was approved by the Ethics and Research Committee of the Huadong Sanatorium Health Examination Center (approval No. ECHS2023-03). Personal information was anonymized to protect personal privacy; statistical analyses were strictly conducted with confidentiality, and the data were only used for scientific purposes. Therefore, the requirement for informed consent was waived.

### Baseline data collection and measurements

Anthropometric measurements, including weight, height, waist circumference (WC), and hip circumference (HC), of all participants were obtained by two well-trained nurses. WC was measured at the midpoint of the costal margin and the iliac crest to the nearest 0.5 cm while the participants gently exhaled. HC was measured by determining the horizontal circumference of the highest point of the hip in the standing position. BMI was calculated using the following equation: weight/[height(m)]^2^. In Chinese adults, normal weight is defined as BMI < 24.0 kg/m^2^ whereas overweight/obesity is defined as BMI ≥ 24.0 kg/m^2^. The waist–hip ratio (WHR) was calculated as WC (cm) divided by HC (cm). The participants’ resting systolic blood pressure (SBP) and diastolic blood pressure (DBP) were measured on the upper arm by an electronic brachial sphygmomanometer (T30J; OMRON, Japan). Regular physical exercise was defined when participants exercised at a high intensity at least 3 times per week or at a moderate intensity at least 5 times per week. Medication treatments including antihypertensive drug and lipid-lowering drug was also collected by trained staff using a standard questionnaire.

Blood specimens (8–10 mL) were collected from the antecubital vein after 12 h of overnight fasting. The serum was placed at room temperature for 30 min and centrifuged at 3,000 rpm for 10 min. The blood indicators included fasting plasma glucose (FPG), triglycerides (TG), total cholesterol (TC), low-density-lipoprotein cholesterol (LDL-C), and high-density lipoprotein cholesterol (HDL-C) levels and white blood cell (WBC), neutrophil (NE), and lymphocyte (LY) counts. All blood samples were tested within 24 h at the medical laboratory center of the Huadong Sanatorium.

Diabetes mellitus was defined by an FPG level of more than 7.0 mmol/L or current intake of antidiabetic medication, and hyperglycemia was defined by a FPG level of ≥ 6.1 mmol/L, while hypertension was diagnosed when blood pressure exceeded 140/90 mmHg or the participants were receiving anti-hypertensive treatment.

### Measurement of body composition using bioelectrical impedance

Assessment of body composition was performed by a professional nutritionist using a segmental multifrequency bioelectrical impedance analysis (BIA) system (InBody 4.0; InBody Co., South Korea). The following validated equation derived by Janssen et al. was used to evaluate the skeletal muscle mass in each participant [21]: skeletal muscle mass (kilograms) = [(height^2^/BIA resistance × 0.401) + (sex × 3.825) + (age × −0.071)] + 5.102, where height was recorded in centimeters, and BIA resistance was recorded in Ohm. For sex, man was coded as 1 and woman as 0; age was recorded in years. The appendicular skeletal mass (ASM) was calculated as the sum of lean muscle mass in both upper and lower limbs. The ASMI was calculated by dividing the ASM by the square of the body height (kg/m^2^). As recommended by the Asian Working Group for Sarcopenia (AWGS) [22], low skeletal muscle mass was defined by an ASMI of ≤ 5.7 kg/m^2^ for females.

### Carotid artery ultrasonography

All participants underwent carotid ultrasonography examinations using a high-resolution B-mode ultrasonographic device (LOGIQ E9; GE, USA) with a 10-MHz linear transducer [23]. Both common carotid arteries were scanned by evaluating the cIMT at three points on the far wall of the middle and distal carotid arteries and 1-1.5 cm proximal to the dilatation of the carotid bulb. Well-trained physicians were responsible for image readings and analysis, and the mean cIMT value of six measurements from both the right and left carotid arteries were used. CAP was defined if the focal wall thickness of a common carotid artery was ≥ 1.5 mm or the focal thickening structure of the surrounding wall was > 50% [24].

### Statistical analysis

Statistical analyses were performed using the SPSS 25.0 and STATA 16.0 software packages. Continuous variables were described as mean ± standard deviation or medians with interquartile ranges and were based on the normality test. Categorical variables were presented as numbers (n) with percentages (%). The baseline characteristics and the biochemical parameters were compared using Student’s t-test or the Mann–Whitney *U* test for continuous variables and the chi-square test for categorical variables. Multivariable logistic regression analysis was performed to determine the relationship between ASMI quartiles and CAP risk. Models were adjusted for age, regular exercise, antihypertensive medication use, lipid-lowering medication use, BMI, WC, WHR, diabetes, hypertension, TG level, WBC and NE counts, and the neutrophil-to-lymphocyte ratio (NLR). Adjusted odds ratios (ORs) were presented with 95% confidence intervals (CIs). Restricted cubic spline regression analysis was performed to estimate the nonlinear association between ASMI and CAP in participants stratified by different BMI classes, and the knots were placed at the 5th, 35th, 65th, and 95th percentiles. Multivariable logistic regression analysis was also performed to evaluate the BMI-specific associations between low muscle mass and CAP risk in individuals with and without hyperglycemia or hypertension. The interactions of the ASMI with different BMI categories among those with and without hypertension or hyperglycemia were assessed by including stratification analysis and interaction tests in the regression model. A two-tailed *P* value < 0.05 was considered statistically significant.

## Results

### Baseline characteristics of the study participants in different BMI categories

**Fig. 1:**
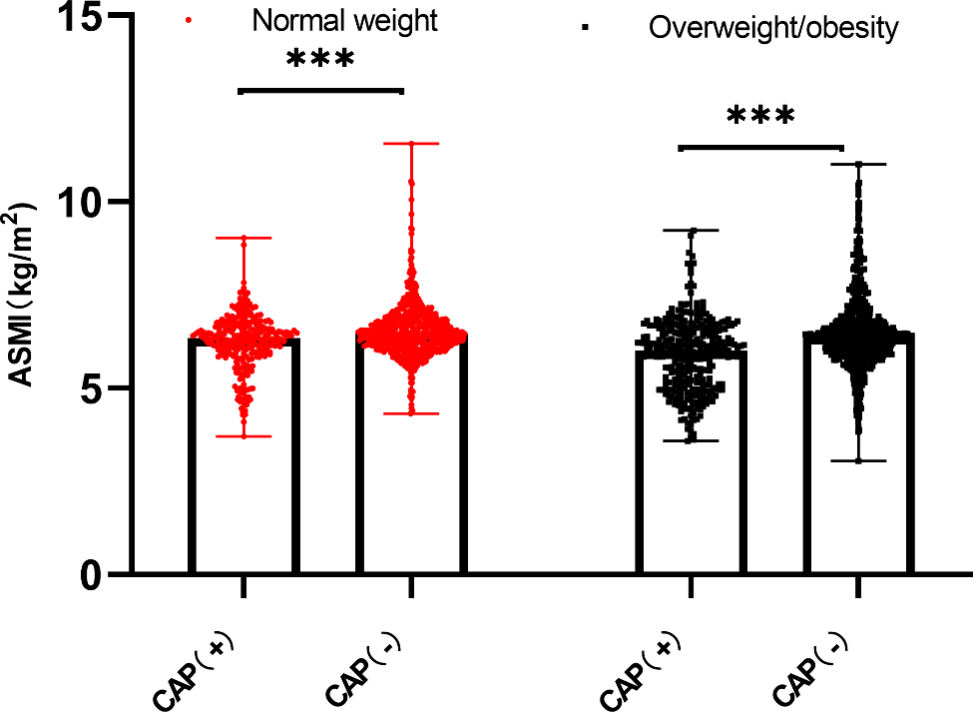
Comparison of ASMI values among groups with normal weight and overweight/obesity; ***, *P* < 0.001

**Fig. 2:**
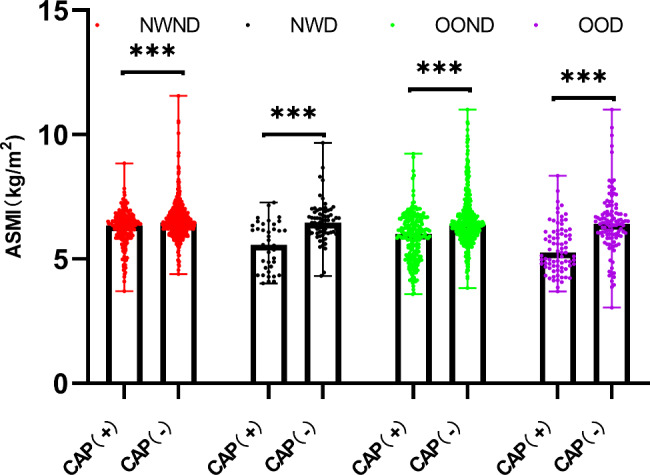
Comparison of ASMI values among groups with and without hypertension (NWNH, normal weight without hypertension; NWH, normal weight with hypertension; OONH, overweight/obesity without hypertension; OOH, overweight/obesity with hypertension); ***, *P*<0.001

**Fig. 3:**
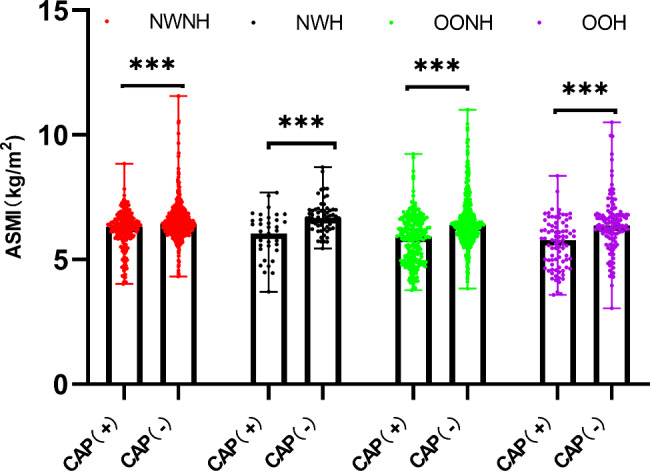
Comparison of ASMI values among groups with and without prediabetes/diabetes (NWND, normal weight without prediabetes/diabetes; NWD, normal weight with prediabetes/diabetes; OOND, overweight/obesity without prediabetes/diabetes; OOD, overweight/obesity with prediabetes/diabetes); ***, *P* < 0.001

Baseline characteristics of all groups classified by BMI are summarized in Table 1. The mean age of the 2048 Chinese postmenopausal women evaluated in this analysis was 57.25 ± 7.54 years. The prevalence of CAP in postmenopausal women was 26.9% (289 women) in the normal-weight population and 32.8% (319 women) in participants with overweight/obesity. In both normal-weight and overweight/obese populations, individuals with CAP tended to be older and had higher WC, WHR, and SBP or a higher prevalence of hypertension and antihypertensive/lipid-lowering medication use (*P*＜0.05). Moreover, normal-weight individuals with CAP showed higher BMI and WBC counts than those without CAP. Individuals with overweight/obesity showed a significantly greater incidence of diabetes, significantly higher DBP and FPG and triglyceride levels, and significantly higher neutrophil counts and NLR values. Comparisons of ASMI values among groups are also shown in Figs. 1, 2 and 3. Participants with CAP showed significantly lower ASMI values than the control group (Fig. 1), while subgroup analyses (Figs. 2 and 3) also indicated that normal-weight or overweight/obese individuals with CAP had lower ASMI values among those with and without hypertension/hyperglycemia (*P* < 0.001).

**Table 1:** Baseline characteristics of the study population according to the presence of carotid atherosclerosis stratified by BMI classification

Variables	Total (n = 2048)	Normal weight (n = 1074)		P value	Overweight/obesity (n = 974)		P value
		CAP (+) (n = 289)	CAP (-) (n = 785)		CAP (+) (n = 319)	CAP (-) (n = 655)	
Age (years)	57.25 ± 7.54	57.71 ± 6.78	56.37 ± 5.85	0.003	58.68 ± 9.70	57.41 ± 8.30	0.034
Regular exercise (n, %)	434(21.2)	36(12.5)	202(25.7)	0.001	41(12.9)	155(23.7)	0.001
BMI (kg/m^2^)	24.09 ± 2.97	22.03 ± 1.33	21.82 ± 1.48	0.027	26.54 ± 2.21	26.52 ± 2.23	0.926
WC (cm)	78.89 ± 8.09	74.99 ± 5.93	73.63 ± 5.29	0.001	85.27 ± 7.12	83.79 ± 6.89	0.002
WHR	0.86 ± 0.18	0.83 ± 0.05	0.82 ± 0.05	0.010	0.92 ± 0.05	0.89 ± 0.30	0.012
ASMI (kg/m^2^)	6.37[5.97, 6.77]	6.34[5.93, 6.65]	6.46[6.16, 6.82]	< 0.001	6.00[5.15, 6.58]	6.39[5.97, 6.81]	< 0.001
FPG (mmol/L)	5.50 ± 1.00	5.45 ± 0.84	5.34 ± 0.95	0.092	5.76 ± 1.22	5.58 ± 0.96	0.022
Diabetes (n, %)	111(5.4)	13(4.5)	28(3.6)	0.476	34(10.7)	36(5.5)	0.005
SBP (mmHg)	121.87 ± 17.92	119.56 ± 18.35	116.29 ± 16.17	0.008	129.42 ± 17.87	125.91 ± 17.43	0.004
DBP (mmHg)	71.94 ± 10.12	70.27 ± 10.43	69.37 ± 9.35	0.177	75.80 ± 10.63	73.88 ± 9.64	0.005
Hypertension (n, %)	318(15.5)	39(13.5)	62(7.9)	0.007	84(26.3)	133(20.3)	0.040
TG (mmol/L)	1.22[0.89, 1.78]	1.11[0.80, 1.64]	1.07[0.81, 1.41]	0.125	1.54[1.07, 2.42]	1.37[0.99, 1.98]	0.007
TC (mmol/L)	5.17[4.59, 5.76]	5.20[4.65, 5.75]	5.20[4.60, 5.84]	0.821	5.06[4.55, 5.64]	5.14[4.58, 5.76]	0.155
LDL-C (mmol/L)	2.35[2.80, 3.87]	3.35[2.85, 3.82]	3.35[2.81, 3.85]	0.949	3.31[2.77, 3.79]	3.35[2.80, 3.91]	0.281
HDL-C (mmol/L)	1.47[1.23, 1.71]	1.50[1.31, 1.79]	1.51[1.30, 1.79]	0.836	1.35[1.17, 1.60]	1.35[1.15, 1.57]	0.380
WBC count (×10^9^/L)	5.43[4.63, 6.41]	5.31[4.50, 6.14]	5.10[4.37, 5.98]	0.025	5.85[4.96, 6.92]	5.79[4.88, 6.76]	0.247
NE count (×10^9^/L)	2.87[2.32, 3.58]	2.77[2.28, 3.44]	2.69[2.16, 3.28]	0.061	3.29[2.58, 4.04]	3.01[2.47, 3.75]	0.002
LY count (×10^9^/L)	2.05[1.72, 2.46]	2.01[1.67, 2.40]	1.91[1.62, 2.34]	0.091	2.11[1.81, 2.56]	2.18[1.83, 2.57]	0.418
NLR	1.40[1.11, 1.77]	1.35[1.06, 1.78]	1.36[1.10, 1.74]	0.980	1.49[1.21, 1.93]	1.38[1.11, 1.75]	0.001
Antihypertensive medication use (n, %)	90(4.4)	22(7.6)	17(2.2)	< 0.001	34(10.7)	17(2.6)	< 0.001
Lipid-lowering medication use (n, %)	160(7.8)	37(12.8)	41(5.2)	< 0.001	43(13.5)	39(6.0)	< 0.001

BMI, body mass index; CAP, carotid artery plaque; DBP, diastolic blood pressure; FPG, fasting plasma glucose; HDL-C, high-density lipoprotein cholesterol; LDL-C, high-density lipoprotein cholesterol; LY, lymphocyte; NE, neutrophil; NLR, neutrophil-to-lymphocyte ratio; SBP, systolic blood pressure; TC, total cholesterol; TG, triglycerides; WC, waist circumference; WBC, white blood cell; WHR, waist–hip ratio

### Baseline characteristics of the study participants in relation to the ASMI quartiles

Table 2 presents the characteristics of normal-weight and overweight/obese participants in relation to the ASMI quartiles. Participants in the lowest ASMI quartiles tended to be younger than those in the highest ASMI quartile in both the normal-weight group (55.80 ± 5.92 years vs. 56.91 ± 6.99 years) and overweight/obese group (56.48 ± 9.31 years vs. 58.28 ± 9.85 years; all *P*_for trend_ < 0.001), and showed significantly higher values for BMI, WC, TG and HDL-C levels, and NE count (all *P*_for trend_ < 0.05) in all groups. Normal-weight participants in the lowest ASMI quartiles had higher WHR values, while the WBC and NLR counts were significantly higher in overweight/obese individuals in the lowest ASMI quartiles than those in the highest ASMI quartile (all *P*_for trend_ < 0.05).

**Table 2:** Baseline characteristics of the study population according to ASMI quartiles stratified by BMI category

Variables	Normal weight (n = 1074)				*P* for trend	Overweight/obesity (n = 974)				*P* for trend
	ASMI Quartiles					ASMI Quartiles				
	Q1 (< 6.10)	Q2 (6.10∼6.42)	Q3 (6.43∼6.79)	Q4 (≥ 6.80)		Q1 (< 5.81)	Q2 (5.81∼6.30)	Q3 (6.31∼6.73)	Q4 (≥ 6.74)	
Age (n, %)	55.80 ± 5.92	56.81 ± 5.60	57.42 ± 5.91	56.91 ± 6.99	0.019	56.48 ± 9.31	58.45 ± 7.57	58.09 ± 8.20	58.28 ± 9.85	0.046
Regular exercise (n, %)	26(13.8)	69(23.1)	76(24.8)	67(24.0)	0.017	36(11.0)	54(24.0)	47(23.7)	59(26.2)	< 0.001
BMI (kg/m^2^)	22.37 ± 1.31	21.75 ± 1.41	21.69 ± 1.47	21.88 ± 1.45	< 0.001	26.93 ± 2.43	26.33 ± 2.03	26.14 ± 1.89	26.49 ± 2.30	0.015
WC (cm)	77.39 ± 4.68	74.51 ± 4.99	72.53 ± 5.08	71.46 ± 5.35	< 0.001	88.92 ± 7.40	84.50 ± 5.62	81.93 ± 5.90	81.72 ± 6.47	< 0.001
WHR	0.84 ± 0.04	0.83 ± 0.05	0.82 ± 0.05	0.82 ± 0.05	< 0.001	0.92 ± 0.06	0.88 ± 0.05	0.91 ± 0.49	0.88 ± 0.05	0.260
FPG (mmol/L)	5.45 ± 0.98	5.33 ± 0.86	5.37 ± 1.09	5.32 ± 0.75	0.182	5.65 ± 0.96	5.75 ± 1.27	5.51 ± 0.80	5.66 ± 1.11	0.462
Diabetes (n, %)	12(4.5)	10(3.6)	12(4.5)	7(2.7)	0.406	18(7.4)	22(9.0)	11(4.5)	19(7.9)	0.680
SBP (mmHg)	117.71 ± 16.61	116.51 ± 15.69	116.45 ± 16.89	118.06 ± 18.16	0.830	129.34 ± 17.16	126.21 ± 18.68	125.29 ± 16.53	127.42 ± 17.98	0.184
DBP (mmHg)	70.52 ± 9.87	69.20 ± 9.09	69.45 ± 10.29	69.27 ± 9.36	0.190	76.42 ± 10.31	73.84 ± 10.35	73.36 ± 9.59	74.40 ± 9.55	0.021
Hypertension (n, %)	23(9.3)	17(6.1)	28(10.6)	31(11.8)	0.128	62(25.4)	50(20.5)	50(20.4)	55(22.8)	0.508
TG (mmol/L)	1.16[0.89, 1.63]	1.06[0.80, 1.42]	1.02[0.76, 1.45]	1.07[0.80, 1.36]	0.010	1.58[1.13, 2.44]	1.34[1.02, 1.92]	1.30[0.94, 1.87]	1.46[1.00, 2.17]	0.001
TC (mmol/L)	5.14[4.56, 5.78]	5.14[4.67, 5.70]	5.29[4.63, 5.89]	5.24[4.69, 5.86]	0.250	5.05[4.49, 5.68]	5.06[4.57, 5.68]	5.17[4.68, 5.78]	5.24[4.58, 5.75]	0.187
LDL-C (mmol/L)	3.33[2.78, 3.80]	3.35[2.88, 3.79]	3.35[2.90, 3.89]	3.35[2.79, 3.91]	0.479	3.31[2.78, 3.87]	3.30[2.82, 3.76]	3.35[2.85, 3.95]	3.32[2.70, 3.89]	0.284
HDL-C (mmol/L)	1.48[1.24, 1.76]	1.50[1.32, 1.74]	1.52[1.32, 1.81]	1.58[1.38, 1.84]	0.004	1.33[1.14, 1.57]	1.33[1.16, 1.50]	1.42[1.22, 1.67]	1.39[1.16, 1.60]	0.034
WBC count (×10^9^/L)	5.28[4.53, 6.09]	5.09[4.34, 5.82]	5.04[4.35, 6.03]	5.18[4.41, 6.21]	0.191	6.11[5.23, 7.20]	5.54[4.74, 6.36]	5.54[4.70, 6.48]	5.94[5.09, 7.00]	< 0.001
NE count (×10^9^/L)	2.83[2.25, 3.46]	2.66[2.18, 3.19]	2.61[2.11, 3.29]	2.80[2.18, 3.50]	0.020	3.36[2.67, 4.03]	2.89[2.42, 3.58]	2.86[2.38, 3.63]	3.18[2.51, 3.89]	< 0.001
LY count (×10^9^/L)	1.96[1.64, 2.37]	1.88[1.64, 2.34]	1.94[1.63, 2.38]	1.94[1.63, 2.34]	0.938	2.20[1.84, 2.60]	2.09[1.76, 2.45]	2.15[1.77, 2.56]	2.19[1.88, 2.68]	0.053
NLR	1.40[1.13, 1.79]	1.34[1.10, 1.67]	1.32[1.03, 1.71]	1.37[1.08, 1.82]	0.121	1.52[1.19, 1.95]	1.39[1.15, 1.72]	1.37[1.11, 1.75]	1.37[1.13, 1.80]	0.029
Antihypertensive medication use (n, %)	14(7.4)	10(3.3)	7(2.3)	8(2.9)	0.016	28(8.6)	9(4.0)	7(3.5)	7(3.1)	0.003
Lipid-lowering medication use (n, %)	24(12.7)	20(6.7)	18(5.9)	16(5.7)	0.010	39(12.0)	17(7.6)	14(7.1)	12(5.3)	0.005

ASMI, appendicular skeletal muscle index; BMI, body mass index; DBP, diastolic blood pressure; FPG, fasting plasma glucose; HDL-C, high-density lipoprotein cholesterol; LDL-C, high-density lipoprotein cholesterol; LY, lymphocyte; NE, neutrophil; NLR, neutrophil-to-lymphocyte ratio; SBP, systolic blood pressure; TC, total cholesterol; TG, triglycerides; WC, waist circumference; WBC, white blood cell; WHR, waist–hip ratio

### Dose-response relationship between ASMI values and CAP risk in postmenopausal women

**Fig. 4:**
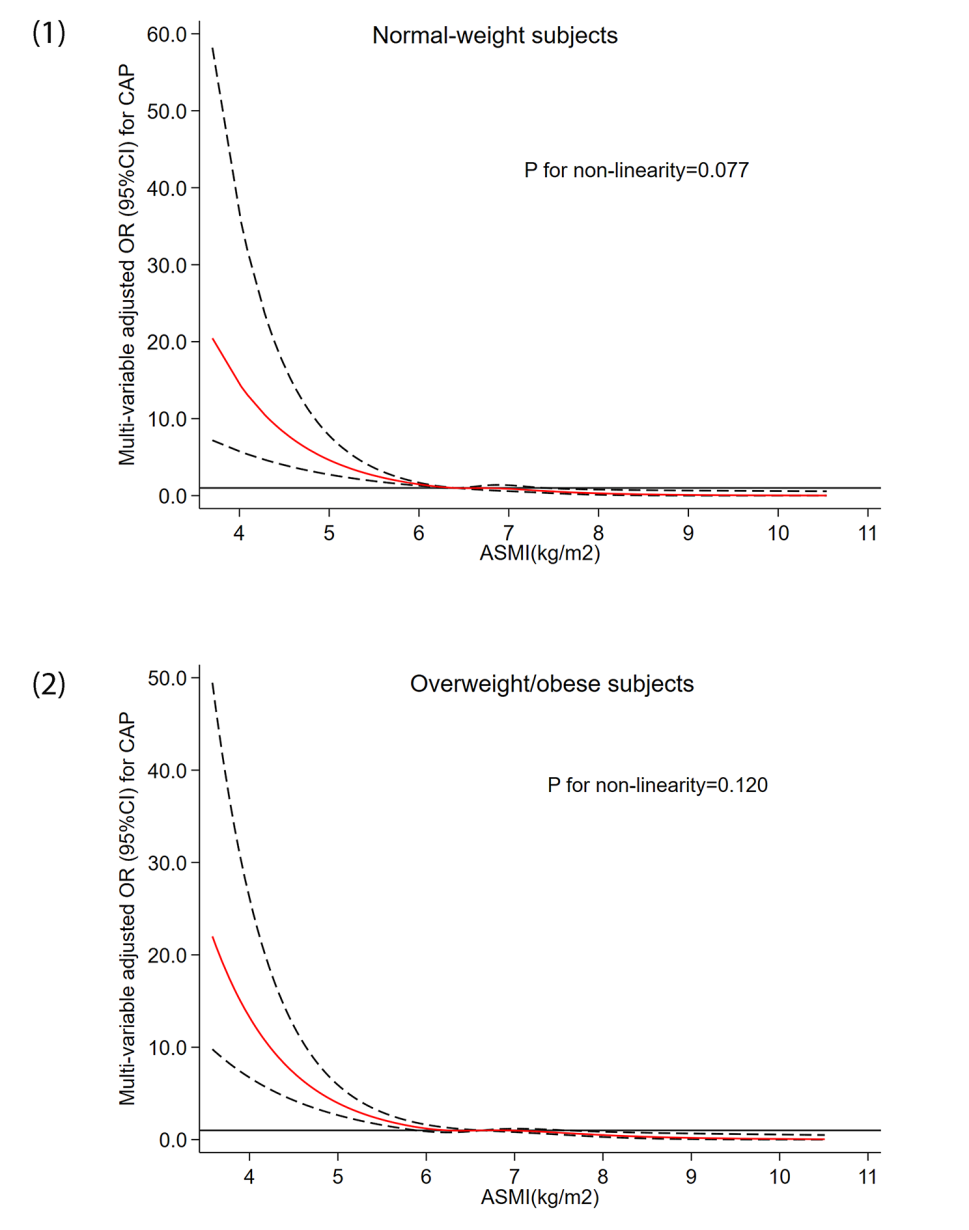
**(1).** Dose-response relationship between ASMI and risk of CAP in postmenopausal women in the normal-weight group. The long solid line indicates that OR is equal to 1, the red line and the area between the short dashed lines indicate ORs and their 95% CIs. **(2).** Dose-response relationship between ASMI and risk of CAP in postmenopausal women in the overweight/obese group. The long solid line indicates that OR is equal to 1, the red line and the area between the short dashed lines indicate ORs and their 95% CIs.

The dose-response relationship between ASMI values and CAP risk stratified by BMI category has been demonstrated in Fig. 4. The horizontal lines represent the 5th, 35th, 65th, and 95th percentiles of ASMI, while the restricted cubic spline regression line (red line) represents multivariable-adjusted ORs for CAP with four knots located at the 5th, 35th, 65th, and 95th percentiles of ASMI. The restricted cubic spline analysis demonstrated that a reduction in the ASMI value significantly increased the risk of developing CAP when ASMI values were below 6.41 kg/m^2^ and 6.26 kg/m^2^ in both normal-weight and underweight/obese individuals after adjusting for confounding factors, including age, BMI, WC, WHR, hypertension, diabetes, TG level, WBC and NE counts, and NLR. Moreover, a nonlinear relationship between ASMI and CAP was not detected in the normal-weight group (*P*_for non−linearity_ = 0.077) and the overweight/obese group (*P*_for non−linearity_= 0.120).

### Association between ASMI quartiles and CAP risk in participants with and without hyperglycemia/hypertension

Normal-weight or overweight/obese postmenopausal women showed an increased risk of CAP with decreasing ASMI quartiles (all *P*_*for trend*_ < 0.001) (Supplementary data). After adjustment for confounding factors in multivariable logistic regression analysis (Table 3), the OR and 95% CI for CAP were 2.43 (1.44 ~ 4.12) for non-hypertensive individuals and 5.90 (1.46 ~ 11.49) for hypertensive individuals among the normal-weight participants in the lowest ASMI quartile. Furthermore, overweight/obese participants in the lowest ASMI quartile showed a significantly higher risk of CAP; this finding was observed in both non-hypertensive individuals (OR = 4.82; 95% CI: 2.79 ~ 8.33) and hypertensive individuals (OR = 7.63; 95% CI: 1.62∼35.86). Moreover, the risk of CAP among non-hyperglycemic individuals was significantly higher in the lowest ASMI quartile (adjusted OR = 2.61 for normal-weight participants and 2.94 for overweight/obese participants) than in the highest ASMI quartile (Table 4). Moreover, the risk for CAP development in the lowest ASMI quartiles was greater than that in the highest ASMI quartiles among hyperglycemic individuals (adjusted OR = 6.66 for normal-weight participants and 8.11 for overweight/obese participants), and the results also indicated that the risk of CAP development in all participants increased linearly with decreasing ASMI quartiles in comparison with the highest ASMI quartiles (all P_for trend_ < 0.001). Besides, with the lowest quartile of ASMI in fully adjusted models, the risk of CAP among overweight/obese subjects was more significant than that among normal-weight subjects with and without hypertension or hyperglycemia (all P for interaction < 0.05).

**Table 3:** Odds ratios for CAP in relation to ASMI quartiles in postmenopausal women with and without hypertension

	Non-hypertensive participants				Hypertensive participants		
	Model 1	Model 2	Model 3		Model 1	Model 2	Model 3
**Normal weight**							
ASMI (continuous variable)	0.66(0.60 ~ 0.73)	0.67(0.59 ~ 0.74)	0.67(0.60 ~ 0.75)		0.58(0.46 ~ 0.71)	0.52(0.41 ~ 0.65)	0.51(0.40 ~ 0.65)
ASMI quartile							
Q1	2.42(1.56 ~ 3.76)^***^	2.33(1.38 ~ 3.94)^**^	2.43(1.44 ~ 4.12)^**^		5.58(2.27 ~ 13.70)^***^	5.24(1.73 ~ 12.48)^**^	5.90(1.46 ~ 11.49)^**^
Q2	1.66(1.06 ~ 2.60)^*^	1.65(1.01 ~ 2.68)^*^	1.72(1.05 ~ 2.81)^*^		1.24(0.28 ~ 5.45)	1.23(0.25 ~ 6.08)	1.40(0.28 ~ 7.11)
Q3	1.37(0.87 ~ 2.16)	1.36(0.85 ~ 2.17)	1.41(0.88 ~ 2.26)		1.63(0.47 ~ 5.71)	1.52(0.40 ~ 5.72)	1.44(0.37 ~ 5.61)
Q4	1.00(reference)	1.00(reference)	1.00(reference)		1.00(reference)	1.00(reference)	1.00(reference)
*P*_for trend_	< 0.001	< 0.001	< 0.001		< 0.001	< 0.001	< 0.001
**Overweight/obese**							
ASMI (continuous variable)	0.45(0.36 ~ 0.55)	0.44(0.35 ~ 0.56)	0.43(0.34 ~ 0.55)		0.47(0.34 ~ 0.66)	0.49(0.35 ~ 0.69)	0.51(0.36 ~ 0.73)
ASMI quartile							
Q1	4.59(2.83 ~ 7.47)^***^	4.74(2.77 ~ 8.13)^***^	4.82(2.79 ~ 8.33)^***^		7.58(2.16 ~ 26.61)^***^	7.48(1.62 ~ 34.39)^**^	7.63(1.62 ~ 35.86)^**^
Q2	2.00(1.23 ~ 3.24)^**^	2.01(1.22 ~ 3.32)^*^	1.84(1.11 ~ 3.07)^*^		2.44(0.96 ~ 6.24)	3.33(1.19 ~ 9.29)^*^	3.89(1.32 ~ 11.47)^*^
Q3	1.05(0.63 ~ 1.76)	1.04(0.62 ~ 1.74)	0.98(0.58 ~ 1.65)		1.24(0.46 ~ 3.35)	1.23(0.43 ~ 3.54)	1.26(0.42 ~ 3.78)
Q4	1.00(reference)	1.00(reference)	1.00(reference)		1.00(reference)	1.00(reference)	1.00(reference)
*P*_for trend_	< 0.001	< 0.001	< 0.001		< 0.001	< 0.001	< 0.001
*P*_*for interaction*_	< 0.001	< 0.001	< 0.001		< 0.001	0.024	0.006

Model 1: Adjusted for age, regular exercise, antihypertensive medication use, lipid-lowering medication use

Model 2: Model 1 + adjustment for BMI, WC, WHR, and diabetes

Model 3: Model 2 + adjustment for TG level, WBC count, NE count, and NLR. *P < 0.05, **P < 0.01, ***P < 0.001

ASMI, appendicular skeletal muscle index; BMI, body mass index; CAP, carotid artery plaque; NE, neutrophil; NLR, neutrophil-to-lymphocyte ratio; TG, triglycerides; WC, waist circumference; WBC, white blood cell; WHR, waist–hip ratio

**Table 4:** Odds ratios for CAP in relation to ASMI quartiles in postmenopausal women with hyperglycemia and without

	Non-hyperglycemic participants				Hyperglycemic participants		
	Model 1	Model 2	Model 3		Model 1	Model 2	Model 3
**Normal weight**							
ASMI (continuous variable)	0.71(0.65 ~ 0.78)	0.70(0.63 ~ 0.78)	0.71(0.63 ~ 0.79)		0.48(0.37 ~ 0.60)	0.44(0.34 ~ 0.58)	0.45(0.35 ~ 0.60)
ASMI quartile							
Q1	2.40(1.56 ~ 3.71)^**^	2.53(1.49 ~ 4.29)^**^	2.61(1.54 ~ 4.43)^**^		8.85(1.69 ~ 46.35)^***^	6.76(1.11 ~ 41.03)^*^	6.66(1.08 ~ 41.10)^*^
Q2	1.51(0.97 ~ 2.36)	1.62(0.99 ~ 2.63)	1.66(1.02 ~ 2.70)^*^		5.25(0.92 ~ 29.98)	3.87(0.59 ~ 25.45)	4.06(0.60 ~ 27.66)
Q3	1.38(0.89 ~ 2.16)	1.44(0.91 ~ 2.28)	1.48(0.93 ~ 2.34)		2.11(0.35 ~ 12.85)	1.83(0.29 ~ 11.54)	1.93(0.30 ~ 12.38)
Q4	1.00(reference)	1.00(reference)	1.00(reference)		1.00(reference)	1.00(reference)	1.00(reference)
*P*_for trend_	< 0.001	< 0.001	< 0.001		< 0.001	< 0.001	< 0.001
**Overweight/obese**							
ASMI (continuous variable)	0.48(0.39 ~ 0.59)	0.51(0.41 ~ 0.63)	0.51(0.41 ~ 0.64)		0.39(0.26 ~ 0.58)	0.32(0.20 ~ 0.50)	0.30(0.20 ~ 0.47)
ASMI quartile							
Q1	3.04(1.91 ~ 4.83)^**^	2.38(1.44 ~ 3.92)^*^	2.94(1.84 ~ 4.70)^**^		6.99(2.38 ~ 20.52)^***^	6.79(2.30 ~ 20.07)^***^	8.11(2.69 ~ 24.49)^***^
Q2	1.80(1.12 ~ 2.91)^*^	1.60(0.98 ~ 2.61)	1.75(1.08 ~ 2.85)^*^		4.58(1.43 ~ 14.67)^**^	4.67(1.44 ~ 15.10)^**^	3.80(1.16 ~ 12.50)^*^
Q3	1.29(0.77 ~ 2.17)	1.27(0.75 ~ 2.14)	1.28(0.76 ~ 2.16)		4.23(1.37 ~ 13.07)^*^	4.05(1.30 ~ 12.58)^*^	3.78(1.20 ~ 11.95)^*^
Q4	1.00(reference)	1.00(reference)	1.00(reference)		1.00(reference)	1.00(reference)	1.00(reference)
*P*_for trend_	< 0.001	< 0.001	< 0.001		< 0.001	< 0.001	< 0.001
*P*_*for interaction*_	< 0.001	< 0.001	< 0.001		< 0.001	0.003	0.007

Model 1: Adjusted for age, regular exercise, antihypertensive medication use, lipid-lowering medication use

Model 2: Model 1 + adjustment for BMI, WC, WHR, and hypertension

Model 3: Model 2 + adjustment for TG level, WBC count, NE count, and NLR. *P < 0.05, **P < 0.01, ***P < 0.001

ASMI, appendicular skeletal muscle index; BMI, body mass index; CAP, carotid artery plaque; NE, neutrophil; NLR, neutrophil-to-lymphocyte ratio; TG, triglycerides; WC, waist circumference; WBC, white blood cell; WHR, waist–hip ratio

### Association between low muscle mass and CAP risk in non-hyperglycemic, hyperglycemic, non-hypertensive, and hypertensive participants in different BMI categories

**Fig. 5:**
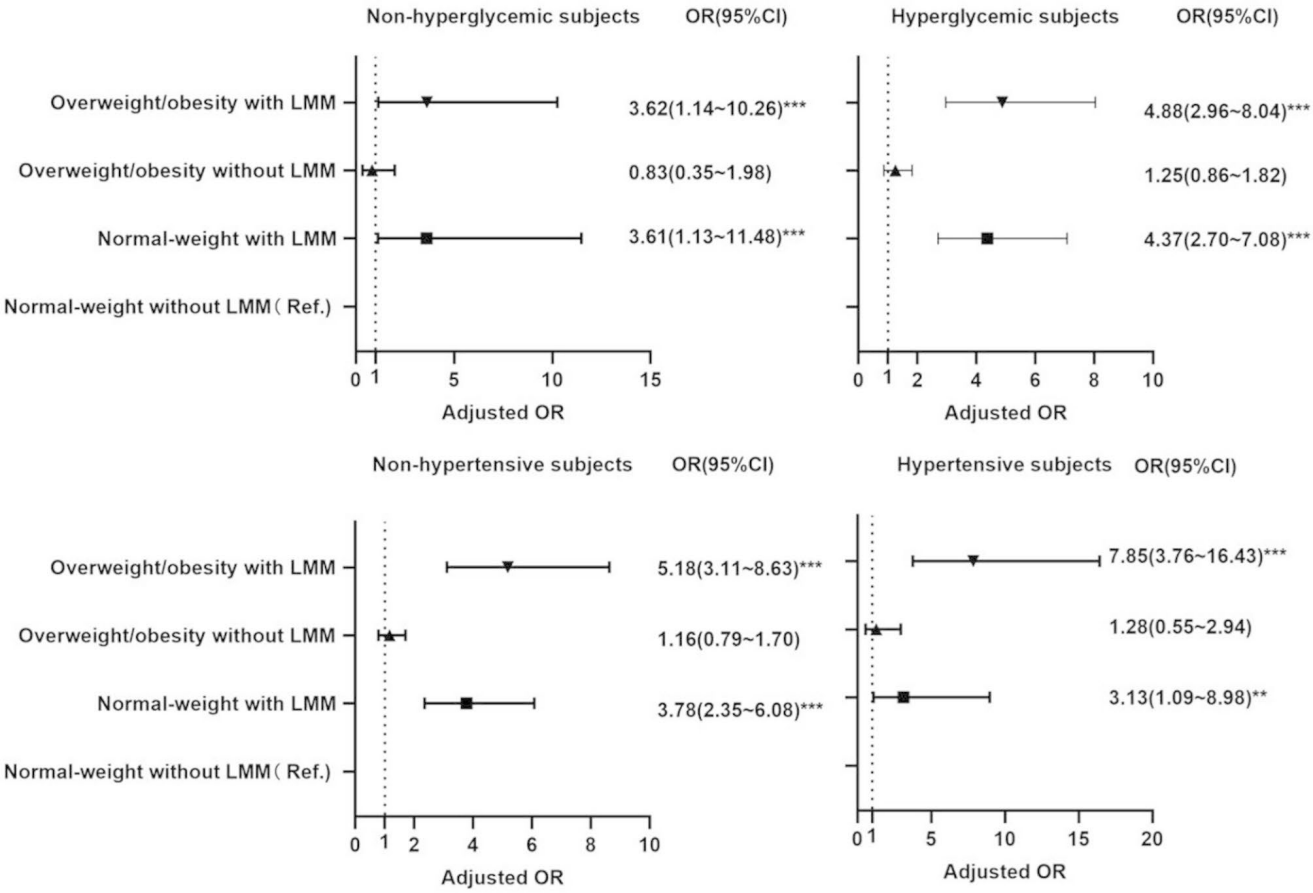
Association between low muscle mass and CAP risk in individuals in different BMI categories stratified by hyperglycemia and hypertension status. **P < 0.01, ***P < 0.001

Multivariable logistic regression analysis demonstrated that low muscle mass significantly increased the risk of CAP development in both normal-weight and overweight/obese individuals, especially in those with hyperglycemia and hypertension (all *P* < 0.01; Fig. 5), while overweight/obese participants with low muscle mass without hypertension or hyperglycemia still showed a higher risk of CAP development (all *P* <0.001).

## Discussion

The current study demonstrated that a lower ASMI is significantly associated with the risk of CAP development in postmenopausal women in different BMI categories. Furthermore, individuals with hyperglycemia or hypertension had higher odds for CAP than those without, and the risk of CAP development was significantly higher in normal-weight or overweight/obese individuals with low muscle mass than in those without, especially in the hyperglycemic and hypertensive populations.

Several studies have reported an inverse association between low skeletal muscle mass and subclinical atherosclerosis [16, 18]. Although a recent study suggested that women with low skeletal muscle mass showed no significantly increased risk of CAP development and being in the highest quartile of cIMT, regardless of BMI [17], this study did not include subgroup analysis according to age groups and had a relatively small sample. Several studies have also indicated that skeletal muscle mass shows an inverse association with cardiovascular disease (CVD) risk in both middle-aged and elderly populations [25, 26], and proven that postmenopausal women are more susceptible to CVD than younger women due to estrogen deficiencies and a dysregulated lipid metabolism [27].

Accumulating evidence has confirmed that estrogen performs a cardioprotective role by directly modulating the renin-angiotensin-aldosterone system [28]. Moreover, since receptors for estrogen and androgens are expressed in both subcutaneous and visceral adipocytes, lipid metabolism in adipose tissues could be affected by excessive changes in the endogenous sex hormone levels in postmenopausal women [29]. Although postmenopausal women show a higher total fat percentage and greater central adipose deposits than premenopausal women, a previous study reported that the testosterone level in postmenopausal women did not differ significantly from that in premenopausal women, suggesting that estrogen decline may primarily account for the sex hormone-related fat redistribution in women after menopause. Additionally, excessive abdominal fat can cause abnormal fatty acid metabolism and lead to dysregulated lipid metabolism, and several cross-sectional studies using dual-energy X-ray absorptiometry (DEXA) have shown that postmenopausal women exhibit lower lean body mass than premenopausal women in the whole body and lower-extremity regions [30], indicating that decreased muscle mass was significantly common in women after menopause. As mentioned above, postmenopausal women may be at a high risk of developing CVD due to the presence of excessive visceral obesity and low skeletal muscle mass. Meanwhile, our study used normal-weight individuals without low muscle mass as a reference group, and the results indicated that low muscle mass can significantly increase the risk of CAP development in normal-weight and overweight/obese individuals, and only overweight/obese individuals without low muscle mass had no CAP risk, which may be explained by the reason that low skeletal muscle mass can lead to more severe metabolic disorders than obesity alone [31]. Cohort research also yielded similar results [32].

Although the mechanism underlying the potential association between low muscle mass and CAP remains unclear, accumulating evidence supports the possibility that low muscle mass and atherosclerosis share common pathophysiologic pathways. Low-grade inflammation in individuals with muscle mass reduction can lead to higher levels of oxidative stress and massive inflammatory cytokine expression that promote endothelial dysfunction and accelerate carotid atherosclerotic formation [33]. This study also suggested that participants in the lower ASMI quartiles showed a linear increase in the neutrophil count in comparison with those in the highest quartile (*P*_for trend_ < 0.001). Insulin resistance is another factor associated with low muscle mass and atherosclerosis. Since skeletal muscle is the primary tissue for peripheral insulin-mediated glucose uptake, insulin resistance simultaneously stimulates protein degradation and gradually induces irreversible muscle mass loss [34]. Insulin resistance also causes abnormal lipid metabolism and endothelial dysfunction through reduced production of nitric oxide, leading to atherosclerosis [35]. Moreover, chronic hyperglycemia promotes the accumulation of advanced glycosylation end products in skeletal muscle, and reductions in skeletal muscle performance, e.g., in grip strength or walking speed, have been reported to be associated with advanced glycosylation end products. Thus, individuals with diabetes are more likely to develop severe muscle loss, and the results of our study confirmed that the risk of CAP development is significantly higher with decreasing ASMI quartiles in hyperglycemic individuals in comparison with non-hyperglycemic individuals. Similarly, the highest odds ratio was nearly 5-fold for the development of CAP in hyperglycemic individuals with low muscle mass. Persistent muscle loss also causes further deterioration of insulin resistance [36, 37]. Moreover, hypertension has been extensively reported to result in increased arterial wall thickness and media-to-lumen ratio [38]. Meanwhile, a systematic review and meta-analysis proved that a reduction in muscle mass is significantly associated with hypertension in older adults [39], and our study also illustrated that a lower ASMI quartile or low muscle mass was associated with higher odds of CAP development in hypertensive individuals than in those without hypertension. In summary, postmenopausal women deserve more attention for prevention of CAP in a hyperglycemic or hypertensive state.

The current study still had several limitations. First, this retrospective study could not confirm a causal association between the ASMI value or low muscle mass and CAP in postmenopausal women. Second, our estimation of skeletal muscle mass did not involve highly accurate modalities such as DEXA, computed tomography, or magnetic resonance imaging. Nevertheless, BIA is recognized as a non-invasive method for assessing skeletal muscle in larger health check-up populations. Previous studies had shown a good linear relationship between BIA and DEXA measurements with a strong correlation coefficient (0.82–0.95). Besides, dietary patterns were not examined, such as whether the participants were vegetarians or those with insufficient protein intake or had a high protein intake or a balanced diet, which may have an impact on muscle mass and/or muscle functions. Finally, the association between low muscle mass and adverse outcomes such as the incidence of CVD was not explored due to the study design, and a prospective cohort study is warranted to investigate the direct association between the relative changes in skeletal muscle mass and the incidence of new-onset cardiovascular events in postmenopausal women.

## Conclusions

In conclusion, low ASMI or skeletal muscle mass was associated with an increased risk of CAP in postmenopausal women with normal weight or overweight/obesity, especially in the hyperglycemic and hypertensive populations. Our findings suggest that the risk of CAP may be modified by skeletal muscle mass maintenance. Additional prospective studies are warranted to prove that increasing ASMI can significantly decrease the CAP incidence in postmenopausal women.

## Electronic supplementary material

Below is the link to the electronic supplementary material.

Supplementary Material 1

## Data Availability

The datasets used and/or analyzed during the current study are available from the corresponding author on reasonable request.

## Abbreviations

ASM: Appendicular skeletal mass
ASMI: Appendicular skeletal muscle index
BIA: Bioelectrical impedance analysis
BMI: Body mass index
CAP: Carotid artery plaque
CI: Confidence interval
CVD: Cardiovascular disease
DBP: Diastolic blood pressure
DEXA: Dual-energy X-ray absorptiometry
FPG: Fasting plasma glucose
HC: Hip circumference
HDL-C: High-density lipoprotein cholesterol
LDL-C: High-density lipoprotein cholesterol
LY: Lymphocyte
NE: Neutrophil
NLR: Neutrophil-to-lymphocyte ratio
OR: Odds ratio
SBP: Systolic blood pressure
TC: Total cholesterol
TG: Triglyceride
WC: Waist circumference
WBC: White blood cell
WHR: Waist–hip ratio
