# Supplementary material for: Low appendicular skeletal muscle index increases the risk of carotid artery plaque in postmenopausal women with and without hypertension/hyperglycemia: a retrospective study
Source: BMC Geriatr. 2023 Jun 20;23:379. doi: 10.1186/s12877-023-04076-w (PMC10283215; doi:10.1186/s12877-023-04076-w)
Supplement: Supplementary file 1 — Supplementary Material 1 [file 12877_2023_4076_MOESM1_ESM.docx]

|  | **Total (n)** | **CAP (n, %)** | **Model 1** | **Model 2** | **Model 3** |
| --- | --- | --- | --- | --- | --- |
| **Normal weight** | 1074 | 289 (26.9) |  |  |  |
| Quartiles of ASMI |  |  |  |  |  |
| Q1 | 269 | 97(36.1) | 3.69(2.40~5.69) | 3.54(2.13~5.88) | 3.65(2.19~6.10) |
| Q2 | 279 | 77(27.6) | 1.38(0.91~2.10) | 1.36(0.86~2.15) | 1.40(0.89~2.22) |
| Q3 | 264 | 65(24.6) | 1.58(1.05~2.37) | 1.57(1.03~2.39) | 1.62(1.06~2.47) |
| Q4 | 262 | 50(19.1) | 1.00 (reference) | 1.00 (reference) | 1.00 (reference) |
| *P* _for trend_ |  |  | ＜0.01 | ＜0.01 | ＜0.01 |
| **Overweight/obese** |  |  |  |  |  |
| Quartiles of ASMI |  |  |  |  |  |
| Q1 | 244 | 125 (51.2) | 4.24(2.81~6.40) | 4.23(2.71~6.59) | 4.14(2.64~6.48) |
| Q2 | 244 | 85(34.8) | 1.56(0.99~2.46) | 1.55(0.98~2.46) | 1.52(0.95~2.43) |
| Q3 | 245 | 57(23.3) | 1.34(0.83~2.16) | 1.30(0.80~2.09) | 1.28(0.79~2.08) |
| Q4 | 241 | 52(21.6) | 1.00(reference) | 1.00(reference) | 1.00(reference) |
| *P* _for trend_ |  |  | ＜0.001 | ＜0.001 | ＜0.001 |

Table S1. Association between ASMI quartiles and CAP risk among normal-weight and overweight/obese postmenopausal women

Model 1: Adjusted for age, regular exercise, antihypertensive medication use, lipid-lowering medication use

Model 2: Model 1 + adjustment for BMI, WC, WHR, diabetes and hypertension

Model 3: Model 2 + adjustment for TG level, WBC count, NE count, and NLR.
